# Millet: a functional powerhouse against chronic lifestyle disease

**DOI:** 10.3389/fnut.2026.1884184

**Published:** 2026-08-07

**Authors:** Suryapratap Ray, Gracy Anu Francis, Sri Sneha Jeyakumar, Pooja Chavan, Srihari Supreethee, Rahul Vashishth

**Affiliations:** 1 School of Biosciences and Technology, Vellore Institute of Technology, Vellore, India; 2 VIT School of Agricultural Innovation and Advanced Learning, Vellore Institute of Technology, Vellore, India

**Keywords:** bioactive compounds, chronic lifestyle diseases, functional foods, millets, nutritional therapeutics

## Abstract

Chronic lifestyle diseases such as cardiovascular disease, type 2 diabetes, and obesity are increasing globally, now accounting for more than two-thirds of deaths worldwide. In this context, millets are regaining attention as nutrient-dense, climate-resilient grains from traditional diets. They are rich in protein, dietary fiber, essential vitamins, minerals, and bioactive compounds that contribute to improved metabolic health and overall well-being. This review compiles and synthesizes current scientific evidence on the nutritional composition, bioactive profile, and disease-modulating mechanisms of millets. It also explores the role of millets in gut microbiome modulation, particularly their prebiotic potential in stimulating the production of short-chain fatty acids (SCFAs) and enhancing gut barrier integrity. Furthermore, the review highlights innovative food applications, including the incorporation of millets into modern food products such as fortified flours, breakfast cereals, snack bars, and beverages. Millets have demonstrated the ability to blunt postprandial blood glucose spikes by up to 15%, reduce cholesterol levels by nearly 10%, and promote natural calorie regulation, thereby supporting weight management and metabolic balance. Their prebiotic fibers nourish beneficial gut bacteria, contributing to improved gut health and immune defense. By integrating traditional knowledge with modern nutritional science, millets emerge as promising functional foods for preventing and managing chronic lifestyle disorders. Promoting millet-based diets offers a sustainable and health-promoting approach to address the growing burden of non-communicable diseases.

## 1 Introduction

Chronic lifestyle diseases (CLDs) are classes of non-communicable conditions like cardiovascular diseases, diabetes, and obesity which are driven by poor diet and low physical activity. These illnesses now account for roughly 71% of all deaths worldwide, highlighting their immense public health impact (1). Diet with rich animal fats and processed sugars, coupled with low intakes of fruits, vegetables, and whole grains, substantially elevate CLD risk (2). Conversely, dietary interventions that lower glycemic index and boost nutrient density through various whole grains and plant-based proteins that have been proven effective in managing diabetes, hypertension, and other lifestyle-linked disorders (3).

Millets (*Pennisetum glaucum*- common pearl millet) are diverse group of small-seeded grasses including sorghum, pearl millet, and finger millet which are gaining renewed interest as functional foods due to their impressive nutrient profiles and long history of cultivation (4, 5). Naturally low on the glycemic index and rich in protein, vitamins, minerals, and dietary fiber, millets have been shown to improve lipid profiles, reduce blood pressure, and significantly lower fasting and post-prandial glucose levels in diabetic individuals (5, 6). Although once dietary staples across Asia and Africa, their popularity decreased with modern agricultural shifts (7). Today, efforts to revive millet consumption underscore both health and socioeconomic gains, yet broader public awareness and further human intervention studies remain essential to fully harness their potential in combatting CLDs (8, 9).

**Figure 1:**
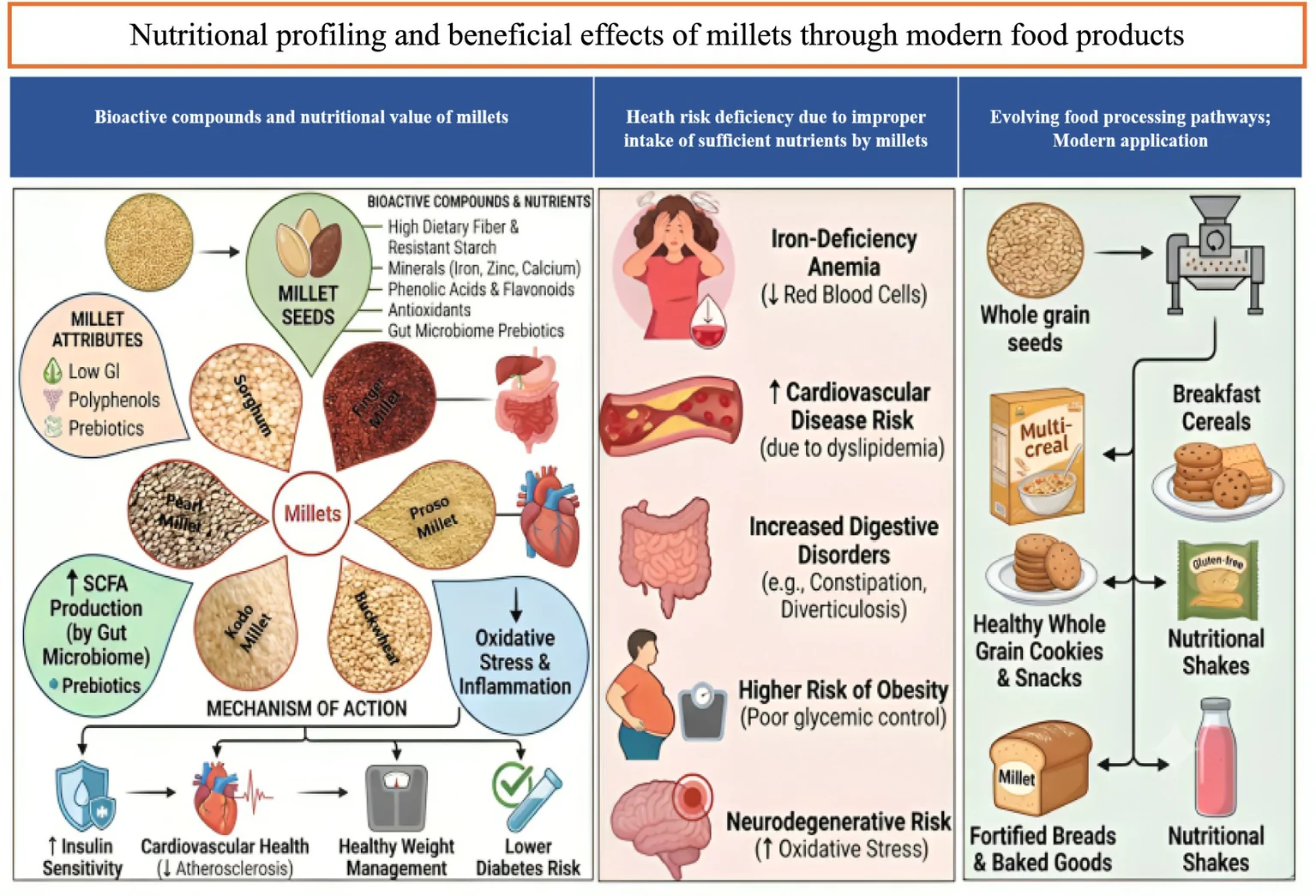
Nutritional and Functional benefits of millets and the evolving food processing products.

Present study explores millet as a promising functional food, particularly in case of CLDs. It systematically addresses millet’s nutritional profile, detailing macronutrients and micronutrients across different millet varieties (Figure 1). A novel perspective is millet’s influence on gut microbiota, which positions millets as potential prebiotic foods capable of promoting beneficial bacterial growth and enhancing gut health, an increasingly relevant aspect given current trends in microbiome research. Additionally, the manuscript delves into in-depth insights on how bioactive compounds present in millet including polyphenols, flavonoids, tannins, and phytosterols might influence disease processes related to diabetes, cardiovascular disorders, obesity, cancer, and neurodegenerative diseases.

A detailed examination of millets within the framework of sustainable food systems and promoting millet cultivation can address nutritional security and environmental resilience amid climate change, resource scarcity, and agricultural challenges is also discussed. Moreover, the manuscript presents current developments in millet-based functional food products, illustrating practical pathways for integrating millets into modern diets through innovative food technologies and novel product formulations. Thus, the manuscript provides a comprehensive, multidimensional evaluation of millets, reinforcing their potential as sustainable, health-promoting food sources with significant implications for global public health and nutrition.

## 2 Nutritional profile of millet

Millets represent a category of diminutive seeds derived from cereal grains, characterized by their high nutritional density and a plethora of health advantages. The grains present a rich composition of carbohydrates (60–70 g), proteins (7.52–12.1 g), fats (3.5–5.2 g), and dietary fiber that supports improved digestive health and the regulation of blood glucose levels (10). Recognized as sustainable crops, millets thrive in arid environments and are endowed with vital amino acids such as cysteine, leucine, lysine, and methionine, in addition to exhibiting a superior mineral profile, including calcium, iron, magnesium, and zinc, compared to other prevalent grains (11). The previously mentioned nutrients are integral to the prevention and management of a range of diseases, encompassing diabetes, cardiovascular disorders, and specific types of cancer. Their low glycemic index is particularly advantageous for individuals diagnosed with diabetes (12). Furthermore, millets possess nutraceutical attributes that facilitate healthy aging, enhance collagen synthesis, and mitigate oxidative stress (13). These types of grains are blended into a wide range of culinary products, which include baked treats, snacks, and drinks, consequently boosting their recognition as beneficial food (14). The antioxidants contained within millets contribute significantly to overall health and longevity by counteracting free radical damage (15).

Additionally, millets hold cultural significance for numerous indigenous communities and are instrumental in sustaining the livelihoods of small-scale agricultural producers (16). Advancing the incorporation of millets into regular dietary practices alongside enhanced research into optimized agricultural techniques and their socio-economic implications could facilitate the establishment of millets as a pivotal element in addressing global food security and nutrition challenges (Table 1).

**Table 1:** Nutritional components of different millets represented in (mg/100 g) (4, 36, 116, 138–140).

Sl. no	Composition	Finger millet (*Eleusine coracana*)	Pearl millet (*Pennisetum glaucum*)	Foxtail millet (*Setaria italica*)	Proso millet (*Panicum miliaceum*)	Kodo millet (*Paspalum scrobiculatum*)	Little millet (*Panicum sumatrense*)	Barnyard millet (*Echinochloa frumentacea*)
1	Protein (g)	7.3	10.6	12.3	12.5	8.3	7.7	11.2
2	Carbohydrate (g)	72.6	67.5	60.9	70.4	65.9	67	65.5
3	Dietary fibre (g)	11.5	11.3	8	7.6	9	7.6	10.1
4	Fat (g)	1.3	5	4.3	1.1	3.6	4.7	3.9
5	Iron (mg)	3.9	8	2.8	0.8	1.7	7.6	12.6
6	Zinc (mg)	2.3	3.1	2.4	1.4	1.5	2.4	2.6
7	Magnesium (mg)	137	137	81	153	119	114	130
8	Calcium (mg)	344	42	31	14	27	17	20
9	Phosphorus (mg)	283	296	290	206	188	220	280
10	Thiamin (mg)	0.42	0.38	0.59	0.2	0.15	0.3	0.33
11	Riboflavin (mg)	0.19	0.21	0.11	0.18	0.09	0.09	0.1
12	Niacin (mg)	1.1	2.8	3.2	4.5	2	3.2	4.2
13	Potassium (mg)	408	307	250	195	144	220	280
14	β-Carotene (μg)	0	0	32	0	0	0	0

### 2.1 Bioactive compounds in millet

Millets contain a variety of bioactive compounds that contribute significantly to the health and nutritional values in humans. These compounds extend beyond basic macro and micronutrients to include various phytochemicals.

#### 2.1.1 Polyphenols

Millets are remarkably abundant in phenolic compounds, exhibiting considerable variation across different varieties. Finger millet, known scientifically as *Eleusine coracana*, has a phenolic composition that varies between 0.3% and 3%, which significantly exceeds the levels typically seen in most cereal grains, as noted by (17). Notable phenolic acids comprise ferulic acid located in pearl millet at 290–711 μg/g, p-coumaric acid in finger millet ranging from 64 to 99 μg/g and foxtail millet at 90–155 μg/g, alongside cinnamic acid found in kodo millet from 35 to 75 μg/g, and chlorogenic acid present in foxtail millet at 42–85 μg/g (18).

The phenolic substances showcase strong antioxidant capabilities, with extracts derived from finger millet illustrating their potential to absorb more than 70% of free radicals in laboratory settings, exceeding the effectiveness of processed wheat and rice (19). The seed coat comprises the majority of phenolic compounds, which help with astringency and provide a shield against oxidative stress and pathogenic entities (20). The DPPH radical scavenging activity in millets varies from 15 to 57 μmol Trolox equivalents/g, with finger millet displaying the highest activity. The anti-inflammatory properties were exhibited by millets due to the presence of phenolics by inhibiting pro-inflammatory cytokines, thereby contributing to the prevention of chronic diseases (21).

#### 2.1.2 Flavonoids

The flavonoid composition in millets constitutes a significant category of bioactive substances known for their beneficial health effects. Specifically, finger millet exhibits an exceptionally elevated flavonoid concentration, approximating 96 mg catechin equivalents per 100 g, which markedly exceeds that found in the majority of other cereal grains (22). In numerous millet varieties, the prominent flavonoids consist of catechin, quercetin, luteolin, and apigenin. Research conducted by (23) established that the flavonoids present in finger millet demonstrate robust antioxidant properties, including the capacity to chelate metal ions and inhibit lipid peroxidation within biological matrices. The genetic variability and cultivation conditions show significant changes, where the concentrations of flavonoids fluctuate between 35 and 68 mg catechin equivalents per 100 g in pearl millet (24). The inhibitory effects were noted on enzymes like *α*-glucosidase and α-amylase, which may facilitate enhanced glycemic regulation and possess anti-diabetic potential from the flavonoids isolated from finger millet (25). Furthermore, studies performed by (26) in both laboratory settings and living organisms revealed that millet-derived flavonoids may impact lipid metabolism, suggesting their potential relevance in controlling dyslipidemia and promoting heart health. It can enhance endothelial functionality and mitigate oxidative stress in vascular tissues (27).

#### 2.1.3 Tannins

Tannins in millets have been regarded as anti-nutritional factors over time, mainly because they can bind to proteins and minerals, which may result in lower levels of nutrient absorption. Of all cereals, finger millet shows the highest tannin content, ranging from 0.04% to 3.47%, and these bioactive compounds are predominantly condensed tannins (proanthocyanidins), with finger millet specifically containing a significant proportion of procyanidin dimers and trimers (28). Empirical research conducted (29) has demonstrated that tannins extracted from finger millet possess robust antimicrobial efficacy against foodborne pathogens, with minimum inhibitory concentrations ranging from 0.5 to 2.0 mg/mL. It has been proposed that potential associations exist between the habitual consumption of tannin-rich foods, including millets, and a diminished incidence of certain malignancies, particularly colorectal cancer. Innovative processing methodologies, such as controlled fermentation and germination, have the potential to enhance tannin concentrations while simultaneously balancing their prospective anti-nutritional attributes with their health-promoting characteristics.

#### 2.1.4 Phytosterols

Millets are characterized by substantial concentrations of phytosterols, which are naturally occurring compounds derived from plants that exhibit structural similarities to cholesterol and have been linked to hypocholesterolemic effects. The pearl millet oil possesses approximately 797 μg/g of total phytosterols, with the predominant components being *β*-sitosterol (447 μg/g), campesterol (75 μg/g), and stigmasterol (54 μg/g). The lipids in proso millet contain phytosterol concentration that ranges from 250 to 400 mg/100 g of oil, which is analogous to the levels observed in widely consumed vegetable oils. The phytosterols extracted from pearl millet can diminish cholesterol absorption in the intestinal tract by competing for absorption sites, which may facilitate the enhancement of lipid profiles (30). The phytosterol extracts from finger millet can inhibit cholesterol micellar solubility by as much as 30%, thereby suggesting a mechanistic pathway for their hypocholesterolemic activities, which is performed under *in vitro* conditions. In addition to their lipid-modulating properties, millet phytosterols have exhibited notable anti-inflammatory effects, revealing a decrease in the expression of inflammatory markers in cell culture models.

### 2.2 Comparative analysis with other grains

A critical evaluation of millet’s nutritional profile in comparison to commonly consumed grains provides compelling evidence for its nutritional superiority in several key aspects. This comparative analysis examines macro and micronutrients, bioactive compounds, and functional properties relative to wheat, rice, quinoa, and other staple grains.

#### 2.2.1 Protein content and quality

Millets exhibit a protein content that is competitive with that of other prevalent cereals (Table 2). Notably, pearl millet (*Pennisetum glaucum*) along with proso millet (*Panicum miliaceum*) shows protein contents from 10% to 12%, which aligns closely with wheat (11%–12%) and is considerably above that of polished rice (6%–7%) (31). Although quinoa shows a marginally elevated protein content (14%–15%), millets offer economic viability and adaptability to challenging agricultural conditions. The evaluation of finger millet’s protein quality via the Protein Digestibility Corrected Amino Acid Score (PDCAAS) indicates a high rating of 0.74, showing it as a superior plant protein source (32). The findings of Vinoth and Ravindhran (149) demonstrate that the essential amino acid profile of finger millet parallels that of egg protein, although lysine is a limiting factor in most cereal grains. For example, barnyard millet exhibits elevated levels of lysine compared to other millet varieties, whereas pearl millet is distinguished by its superior sulfur amino acid content, as noted by (33).

**Table 2:** Overview of the bioactive compounds, health-promoting properties, processing effects, challenges, and future perspectives of Millet.

Category	Component/aspect	Major compounds or features	Health benefits/functions	References
Bioactive compounds	Phenolic compounds & flavonoids	Ferulic acid, caffeic acid, quercetin, catechins, gallic acid	Strong antioxidant activity; scavenges free radicals; reduces oxidative stress and inflammation; protects against chronic diseases	(64, 127)
	Dietary fibers	Soluble and insoluble fibers, resistant starch	Lowers glycemic index; improves blood glucose regulation; enhances satiety; reduces metabolic disorders	(141, 142)
	Phytochemicals	Saponins, tannins, glycosides, phytosterols	Anti-cancer, anti-diabetic, cholesterol-lowering, antimicrobial and antioxidant activities	(65, 143)
Health benefits	Antioxidant activity	Phenolics, flavonoids, tannins	Neutralizes reactive oxygen species (ROS), reduces oxidative damage and inflammation	(65, 95, 97)
	Anti-inflammatory effects	Polyphenols and flavonoids	Suppresses inflammatory mediators and protects tissues from chronic inflammation	(99, 144)
	Cardiovascular protection	Dietary fiber, phytosterols, phenolic compounds	Improves lipid profile; lowers LDL cholesterol; reduces cardiovascular disease risk	(10, 79, 144)
	Diabetes management	Dietary fiber, resistant starch, phenolics	Delays carbohydrate digestion, improves insulin sensitivity, regulates blood glucose levels	(10, 141)
	Cancer prevention	Polyphenols, flavonoids, saponins	Induces apoptosis, inhibits tumor growth, reduces oxidative DNA damage	(87, 89, 144, 145)
	Gut health	Dietary fiber, resistant starch, polyphenols	Promotes beneficial gut microbiota, improves intestinal health and digestive function	(39, 67, 146)
	Antimicrobial activity	Tannins, saponins, phenolic acids	Inhibits growth of pathogenic microorganisms and supports immune health	(120, 143)
Processing & bioavailability	Germination/malting	Activates endogenous enzymes	Increases total phenolic compounds, antioxidant capacity, vitamin content and mineral bioavailability	(117)
	Dehulling	Removal of bran layer	May reduce phenolics and dietary fiber due to loss of outer grain layers	(117, 123)
	Novel processing technologies	Ultrasound, microwave, extrusion	Enhances extraction efficiency, bioavailability and retention of bioactive compounds	(117)
	Fermentation	Lactic acid bacteria and yeast fermentation	Improves nutrient digestibility, mineral bioavailability, probiotic potential and antioxidant activity	(23, 147)
Challenges	Consumer acceptance	Taste, texture and cooking preferences	Limits wider dietary adoption despite health benefits	(141)
	Anti-nutritional factors	Phytates, tannins, enzyme inhibitors	Reduce mineral absorption and nutrient bioavailability; can be minimized by processing methods	(145, 147, 148)
Future Perspectives	Processing innovations	Germination, fermentation, biofortification, advanced processing	Enhance nutritional quality, improve bioavailability and increase consumer acceptance	(117, 123, 141)
	Sustainable food security	Climate-resilient crop with high nutritional value	Helps combat malnutrition, chronic diseases and climate-related agricultural challenges	(141)

#### 2.2.2 Mineral content

Finger millet (*Eleusine coracana*) contains calcium levels that are 5–30 times greater (344 mg/100 g) than those found in other cereals, including wheat (30 mg/100 g), rice (10 mg/100 g), and even quinoa (47 mg/100 g) (34). This exceptionally high calcium concentration renders finger millet a valuable resource for combating calcium deficiency, particularly within populations that exhibit limited dairy intake. Clinical investigations conducted by (35) have revealed significant enhancements in bone mineral density among postmenopausal women who consistently consume finger millet-based diets in comparison to those adhering to rice-based diets. Iron content varies significantly across millet species, with pearl millet providing approximately 8 mg/100 g, which is higher than that of rice (0.7 mg/100 g) and comparable to that of quinoa (8.5 mg/100 g) (36).

Biofortification has further increased the iron content in certain pearl millet varieties to 9–10 mg/100 g through conventional breeding methodologies. The zinc content in millets similarly compares favorably to other grains; for instance, pearl millet contains 3.1 mg/100 g of zinc, in contrast to wheat’s 2.4 mg/100 g and rice’s 1.3 mg/100 g. Phosphorus content in millets (296 mg/100 g in pearl millet) surpasses that of rice (160 mg/100 g) and is comparable to that of wheat (298 mg/100 g). Furthermore, magnesium concentrations in most millet varieties (114 mg/100 g in pearl millet) significantly exceed those in polished rice (25 mg/100 g) and refined wheat flour (22 mg/100 g), thereby contributing to millet’s potential role in the management of chronic conditions such as hypertension and type 2 diabetes.

#### 2.2.3 Dietary fiber

The fiber composition of millets signifies a notable value in their nutritional value with widely consumed refined cereals. Barnyard millet (*Echinochloa frumentacea*) exhibits a dietary fiber content ranging from 9% to 13%, which is significantly higher than that of polished rice (0.2%–0.5%) and comparable to whole wheat (11%–13%) (37). A majority of millet cultivars exhibit dietary fiber levels between 7% and 15%, characterized by a favorable ratio of soluble to insoluble fractions. Studies performed by (38) have demonstrated that the soluble fiber component of finger millet, at 1.8% to 2.2%, is linked to its cholesterol-lowering effects, with clinical trials indicating a reduction of 5% to 8% in serum cholesterol after consistent consumption for 12 weeks. Millet contains a fiber composition that includes *β*-glucans, arabinoxylans, and resistant starch, which enhances the prebiotic functionalities. The dietary fiber derived from millet enhances the production of short-chain fatty acids within the colon, with notable elevations in butyrate, which is beneficial for digestive health (39).

#### 2.2.4 Glycemic index and metabolic responses

Millets are generally characterized by lower glycemic indices in contrast to refined wheat and rice products, thereby presenting a considerable nutritional benefit in a context marked by rising incidences of metabolic disorders. Clinical investigations by documented glycemic indices for finger millet preparations ranging between 54 and 68, for pearl millet between 55 and 65, and foxtail millet between 50 and 60, in comparison to white rice (GI ≈ 73–89) and white wheat bread (GI ≈ 70–75). The lesser impact on blood sugar levels from millets can be explained by their superior ratio of amylose to amylopectin, along with abundant fiber and certain anti-amylase substances, including polyphenols and tannins. Studies conducted indicated that replacing 50% of rice with finger millet in the dietary regimen of type 2 diabetic subjects led to substantial reductions in postprandial glucose (22%) and insulin responses (30%) when contrasted with rice-only meals (5).

#### 2.2.5 Antioxidant activity

In millet, Antioxidant characteristics show significant benefits when compared to conventional cereals. Research conducted by (40) has established that the overall antioxidant activity of finger millet is approximately 3–15 times greater than that of wheat and rice, as assessed by ORAC (Oxygen Radical Absorbance Capacity) metrics. This remarkable antioxidant potential has been ascribed to the synergistic interactions of various bioactive constituents, encompassing phenolic acids, flavonoids, and phytosterols. Fermented millet products show the free radical scavenging activity, with research conducted by (41) resulting in 30%–50% increased antioxidant activity after lactic acid fermentation, a phenomenon not observed to the same degree in fermented wheat or rice products. The allocation of antioxidant compounds within millet grains is distinct from that found in wheat; while significant antioxidants are predominantly located in the bran fraction of wheat, millets contain considerable quantities in the endosperm as well, facilitating the preservation of antioxidant activity even in decorticated or refined millet flours.

#### 2.2.6 Gluten content and allergenicity

All varieties of millet are gluten-free, making them good alternatives for individuals with celiac disease or non-celiac gluten sensitivity. This characteristic represents a considerable advantage over wheat, barley, and rye, which are associated with gluten proteins implicated in the pathogenesis of celiac disease. Clinical investigations have shown the safety and tolerability of millets in patients with celiac disease, revealing no adverse immunological reactions or gastrointestinal symptoms following consumption. Furthermore, millets have nutritional benefits over certain other gluten-free alternatives, such as refined rice products, especially concerning protein content, micronutrient levels, and bioactive compounds.

Millet-based gluten-free products possessed significantly higher protein content (8%–12%) in comparison to rice-based alternatives (4%–7%) and exhibited protein levels comparable to those of quinoa-based products (9%–14%). Additionally, the balanced amino acid composition of millet proteins offers advantages over corn-based gluten-free products, which are significantly deficient in lysine. The minimal cross-reactivity between millet proteins and prevalent food allergens suggests their potential appropriateness for individuals with multiple food allergies, although further comprehensive clinical research is necessary (42).

### 2.3 Emerging research on millet’s micronutrient bioavailability

Emerging investigations regarding the bioavailability of micronutrients within millet underscore the intricate nature of nutrient absorption and the progressive methodologies being examined to address conventional constraints. Rich in iron, zinc, calcium, and selenium, millets face challenges due to anti-nutritional components such as phytic acid, tannins, and dietary fiber, which considerably hinder their bioavailability through the formation of insoluble complexes or by limiting nutrient access (28, 43). Germination and fermentation have demonstrated substantial potential, with a reduction of 50%–60% in phytic acid and an increase of 2–3-fold in the bioavailability of iron and zinc (44). Germination triggers the activation of endogenous phytases, whereas fermentation introduces microbial enzymes and organic acids that enhance the solubility of nutrients (45).

Thermal processing methods, including popping and pressure cooking, disrupt anti-nutrient complexes and cause mineral extraction (5, 46). Initiatives in agronomic and genetic biofortification have further stimulated advancements within this domain, with zinc- and iron-enriched varieties of pearl millet demonstrating marked improvements in micronutrient content and absorption during human trials (47, 48). Moreover, the gut microbiome shows that prebiotics obtained from millet stimulate bacterial development, thus enhancing nutrient metabolism and absorption (39).

The bioactive substances found in millet affect gene regulation related to glucose processing and the body’s antioxidant systems, suggesting that these factors, along with nutritional habits and genetics, are crucial for health benefits (49). Human trials show that optimized consumption of millet leads to increased hemoglobin, ferritin, and zinc levels, as well as enhanced bone density while facilitating glycemic control (50). Collectively combining processing innovations, biofortification strategies, modulation of the gut microbiome, and personalized nutrition through nutrigenomics is unlocking the comprehensive nutritional potential of millets, paving the way in promoting sustainable health.

## 3 Millet and gut microbiome

A well reported area in gut microflora stimulation through millets highlights the prebiotic activities. Study showed the enhanced growth of beneficial gut bacteria due to the consumption of Millete based beverages. Millets contain several prebiotic compounds such as arabinoxylans, inulin, and xylooligosaccharides, which are isolated in the bran and seed coat. These compounds are known to support the growth of beneficial gut bacteria, thereby promoting gut health. The high dietary fiber content in millets contributes to digestive health by supporting regular bowel movements and fostering a healthy gut microbiota. This fiber content is crucial for maintaining a balanced gut environment and preventing constipation (49). Millets are rich in phenolic compounds, which possess antioxidant properties. These antioxidants may help protect gut cells from oxidative damage and reduce inflammation, further supporting overall gut health and well-being (49).

Despite their health benefits, the bioavailability of bioactive compounds in millets can be limited due to the presence of antinutrients. However, appropriate processing techniques can enhance the availability of these nutrients, thereby maximizing their health benefits. The integration of millets into regular diets can be challenging due to their low sensory acceptance and the presence of antinutritional factors. However, advancements in processing technologies and value addition can improve their acceptability and nutritional profile (49). Millets offer significant potential for enhancing gut health due to their prebiotic properties and high fiber content. Future research could focus on conducting well-designed clinical trials to further elucidate the specific effects of millets on the gut microbiome and chronic diseases.

## 4 Mechanistic insights on how millet fights chronic lifestyle diseases

**Figure 2:**
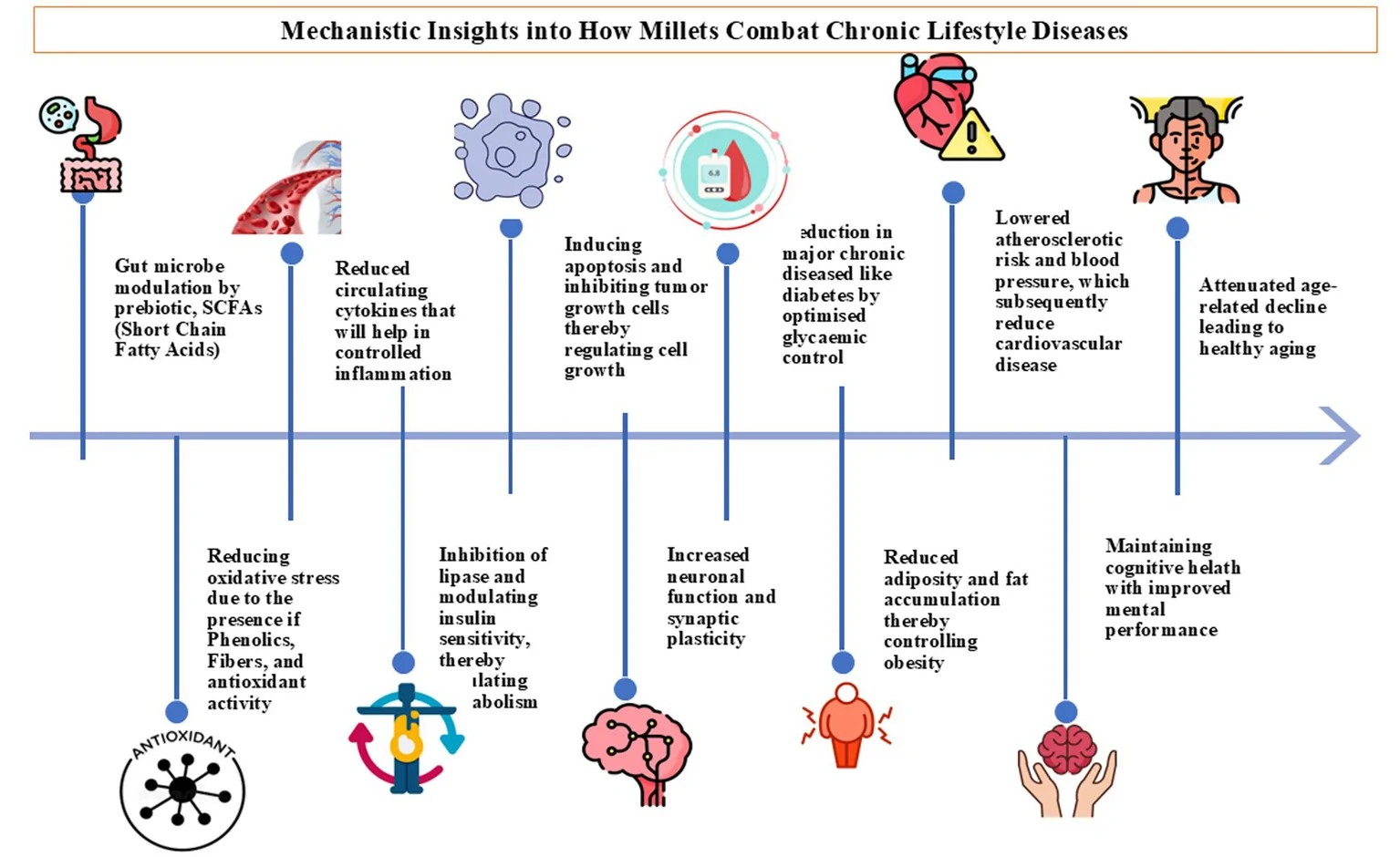
Mechanisms on how millets fight chronic lifestyle diseases.

As discussed above, millet is a nutrient-rich grain that offers potential benefits in preventing and managing various diseases. Below, we discuss how millet influences cardiovascular disease, obesity, cancer, and neurodegenerative disorders through anti-inflammatory and antioxidant effects. How millet consumption reduces the risk of chronic lifestyle diseases are illustrated in Figure 2.

### 4.1 Diabetes and metabolic syndrome

Diabetes Mellitus, along with Metabolic Syndrome (MetS), is a complicated dynamic that profoundly affect the health outcomes worldwide. Those diagnosed with Type 2 Diabetes Mellitus (T2DM) encounter a metabolic obstacle represented by ongoing high blood sugar concentrations, stemming from a poor reaction to or generation of insulin, which ultimately restricts the body’s efficiency in processing carbohydrates, fats, and proteins, resulting in lasting organ issues. The notion of Metabolic Syndrome is characterized by a grouping of metabolic malfunctions, such as central obesity, hypertension, lipid irregularities, and insulin insensitivity, which in combination increase the susceptibility to T2DM and cardiovascular issues (51–53).

Millets releases glucose more slowly than other common staples such as rice and wheat, since they possess low glycemic index (GI), which helps maintain stable blood sugar levels and reduces the risk of glucose spikes in individuals with diabetes (54, 55). A systematic review and meta-analysis reported that regular millet consumption lowers fasting blood sugar by 11.8% and post-prandial glucose by 15.1%, underscoring its practical benefit for glycemic control (56). In direct comparison, millets exhibit a mean GI roughly 36% lower than milled rice and refined wheat, making them a clear advantage for blood sugar management (56). The gradual release of glucose is predominantly influenced by the nutritional composition of millets. Their substantial dietary fiber content not only retards the digestion and absorption of carbohydrates but also enhances satiety, thereby aiding in the regulation of overall caloric consumption (57, 58). Simultaneously, millets exhibit a substantial abundance of phenolic flavonoids and an array of antioxidants that mitigate oxidative stress, a well-documented contributor to insulin resistance, thereby promoting more advantageous insulin signaling pathways (59). Additionally, foxtail millet have revealed their potential to favorably impact gut microbiota, resulting in a growth of *Lactobacillus* populations connected to improved glucose metabolism (60).

Substituting rice-based diet with millet-based alternatives has been demonstrated to markedly diminish postprandial glycemic responses in individuals diagnosed with diabetes, thereby providing a readily applicable intervention for healthcare professionals and nutritionists. In addition to primary dishes, the innovation of low-glycemic index millet snacks offers practical options for individuals with a mobile lifestyle who aspire to uphold metabolic well-being (50). Although these results are persuasive, it is crucial to acknowledge that millets exhibit optimal efficacy when incorporated within a comprehensive dietary framework and a healthy lifestyle. Factors such as portion control, timing of meals, and the overall nutritional composition will exert considerable influence on personal health outcomes. Subsequent investigations, especially long-term clinical studies across diverse population, will be required for refining serving guidelines and validating enduring benefits, thereby ensuring that millet-based nutritional approaches can be advocated with assurance for the prevention and management of diabetes and metabolic syndrome.

In diabetic mouse models, millet-derived phenolic acids from foxtail millet bran have been shown to enhance insulin sensitivity by non-competitively inhibiting *α*-glucosidase activity and blocking the miR-1-3p/PTP1B signalling axis, thereby reducing negative regulation of the insulin pathway in diabetic mice (61). In parallel, millet phenolics exert potent antioxidant and anti-inflammatory effects, which help to mitigate oxidative stress and chronic inflammation, both of which are major contributors to insulin resistance and *β*-cell dysfunction (62). Proteins isolated from foxtail millet, whether raw or cooked, further support glucose homeostasis and β-cell protection. In diabetic mouse models, these protein isolates improved glucose intolerance and insulin resistance by modulating gut microbiota and activating key intracellular signaling cascades such as the GLP-1R/PI3K/AKT pathway, essential for insulin secretion and β-cell survival (63).

Heat-treated millet proteins have also been found to increase the abundance of beneficial bacterial genera like Lactobacillus and Bifidobacterium, reinforcing the gut–pancreas axis and promoting systemic insulin responsiveness (62). At a mechanistic level, foxtail millet supplementation robustly stimulates the PI3K/AKT signaling pathway, shifting metabolism toward glycolysis and suppressing gluconeogenesis, which collectively enhances insulin sensitivity and guards against *β*-cell stress (60). Complementing this, millet-derived compounds inhibit negative regulators of insulin signaling such as protein tyrosine phosphatase 1B (PTP1B), further sustaining insulin receptor activity and preserving β-cell function under metabolic challenge (61). While the molecular elucidations underscore the potential efficacy of millet-derived bioactives in the management of diabetes, the applicability in practical settings is contingent upon various determinants, including the bioavailability of phenolic compounds and proteins, which may be influenced by agricultural methodologies and food processing techniques (54, 62, 64).

### 4.2 Cardiovascular diseases (CVDs)

Rich in phenolic compounds, millets demonstrate strong antioxidant capabilities that counteract reactive oxygen species (ROS) and mitigate oxidative stress, a crucial element contributing to cardiovascular ailments (62, 65). Experimental studies have demonstrated that, bioactive peptides extracted from red millet yellow wine have been demonstrated to activate the Sirt1/Nrf2 signaling pathway, thereby enhancing the intrinsic antioxidant mechanisms of the heart and safeguarding cardiac cells from oxidative injury (66). While these findings highlight a clear protective mechanism, it remains to be verified how effectively these peptides are absorbed and remain active in human circulation. Studies shows that millet’s phytochemicals, particularly those obtained from the outer husks, are crucial elements in lessening inflammation by blocking pro-inflammatory cytokines such as IL-1β and TNF-*α*, along with curtailing the NF-κB signaling pathway (61, 67). Additionally, the polyphenolic agents present in millet bran impact the ROS/miR-149/Akt/NF-κB signaling route, showcasing a dual mechanism that deals with both oxidative stress and inflammatory processes (61). However, the degree to which these anti-inflammatory benefits translate into measurable improvements in human cardiovascular markers requires more targeted clinical research.

The consistent use of millet across different culinary creations indicates a correlation with enhanced lipid statistics, distinguished by reduced total cholesterol, triacylglycerol, and LDL-C concentrations, together with an uptick in HDL-C, which may support the management of hyperlipidemia, a prominent player in cardiovascular problems (68, 69). Some trials have also reported accompanying decreases in BMI and blood pressure, further underscoring millet’s role in overall cardiovascular health (69).

The polyphenolic agents derived from millet husks seemingly provide a shield against atherosclerosis by reinforcing the gut barrier and adjusting the microbiome, which ultimately reduces plaque formation (67). Furthermore, these identical polyphenols impede the formation of foam cells, an initial and pivotal occurrence in the progression of arterial plaque, by restricting lipid absorption and the release of inflammatory mediators in macrophages (70). Despite this promising data, variations in phenolic content caused by agricultural practices and processing methods could significantly influence outcomes. Future work should therefore explore breeding or biotechnological strategies to standardize and enhance millet phenolics, as well as investigate their incorporation into functional foods and nutraceuticals for consistent cardiovascular benefits (16).

Millets have emerged as promising dietary allies in managing chronic lifestyle diseases through their favorable effects on lipid metabolism. Consuming millet consistently has revealed a relationship with remarkable declines in overall cholesterol (TC) and low-density lipoprotein cholesterol (LDL-C), underscored by a comprehensive analysis and meta-study that noted average drops of 8.0% in TC and 10% in LDL-C over intervention spans of 3 weeks to 4 months (69). Another investigation found a standardized mean reduction of 0.60 in LDL-C compared to other staple grains (68). While these findings highlight millets’ cholesterol-lowering potential, most trials have been short-term and conducted in specific populations, underscoring the need for longer, more diverse human studies to confirm sustained benefits.

Beyond lowering “bad” cholesterol, millets also promote higher levels of high-density lipoprotein cholesterol (HDL-C), often dubbed “good cholesterol.” *In vivo* animal studies demonstrate that proso millet protein elevates HDL-C without adversely affecting LDL-C (71), and millet shell polyphenols increased HDL-C in ApoE−/− mice, contributing to atherosclerosis prevention (70). Additionally, foxtail millet supplementation reduced triglycerides by 14.0% in rat models (150), a result echoed in other rodent studies on millet-based diets (72). However, translating these lipid improvements from animals to humans remains an open question that future clinical trials should address.

Polyphenols from millet shells inhibit foam cell formation, a key event in atherosclerosis, by blocking lipid uptake and lowering inflammatory markers (70). Meanwhile, millet proteins appear to modulate gene expression related to lipid metabolism, downregulating lipogenesis and upregulating lipolysis in models like hog millet (72). In head-to-head comparisons with pharmaceuticals, foxtail millet improved lipid profiles though to a lesser extent than atorvastatin, suggesting that millets may serve best as complementary interventions alongside standard therapies (73). This complementary role, however, should be defined through trials that examine millets in combination with lipid-lowering medications.

Integrating millets into a balanced diet offers a holistic approach to hyperlipidemia management, but real-world effectiveness depends on broader dietary and lifestyle factors. Portion size, meal composition, cooking methods, and individual metabolic variability will all influence outcomes. Moreover, agricultural practices and processing techniques can alter millet’s bioactive profile, affecting its lipid-modulating capacity. In order to comprehensively exploit the potential of millet, subsequent research endeavors must encompass prolonged, rigorously controlled human studies involving a variety of demographic cohorts, in addition to explorations into the most effective processing techniques and the development of millet-derived functional food formulations.

### 4.3 Obesity

Millets are abundant in dietary fiber, a component that augments gastric volume and extends the duration of gastric emptying, thereby contributing to heightened feelings of satiety and diminished sensations of hunger. This mechanism helps in lowering overall calorie intake, which is crucial for weight management (56, 74). Moreover, the high protein content in millets contributes to maintaining lean muscle mass and regulating blood sugar levels, further aiding in appetite control and reducing the risk of obesity (75). It is vital to note that the bulk of these interpretations is derived from studies marked by either temporary periods or observational frameworks. The extent to which different millet varieties and individual metabolic responses influence these outcomes remains underexplored, and some studies report inconsistent effects on satiety depending on preparation methods and portion sizes.

Within the framework of glycemic oversight, millets display a low glycemic index, thereby helping to reduce blood glucose levels after meals and enhancing insulin responsiveness. This property is beneficial in managing obesity by preventing rapid spikes in blood sugar and subsequent hunger pangs (76, 77). Multiple research findings imply that millets could support the optimization of lipid profiles, potentially decreasing total cholesterol, triglycerides, and LDL cholesterol, while also fostering higher HDL cholesterol levels. Such changes are instrumental in lowering the chance of heart-related diseases tied to obesity (5, 68). Nevertheless, it is imperative to note that numerous clinical trials employ limited sample sizes or lack comprehensive long-term follow-up, thereby complicating the ability to formulate conclusive assertions regarding the enduring metabolic advantages of millets across varied populations.

Beyond macronutrient composition, bioactive compounds in millet exhibit promising anti-obesity effects. Molecular docking studies have shown that, certain phytochemicals show superior binding energies to the fat mass and obesity-associated (FTO) protein compared to standard anti-obesity drugs like orlistat, suggesting potential as natural inhibitors with fewer side effects (78). Millets possess remarkable properties that combat oxidative stress and inflammation, aiding in the control of obesity, conditions often linked to metabolic issues (74, 79). Additionally, millets function as prebiotics, exerting a favorable influence on gut microbiota and resulting in the augmented synthesis of short-chain fatty acids; these metabolites bolster gut barrier integrity and diminish inflammation, thereby contributing to enhanced metabolic health and effective weight (79).

The high fiber content in millets also prolongs satiety by increasing gastric volume and delaying gastric emptying, leading to reduced overall caloric intake, an essential factor in weight management and obesity prevention (68). Millets’ ability to curb hunger can lower the hunger index, making them an appealing option for people trying to manage their weight (56). Moreover, millets possess anti-inflammatory and antioxidant properties, which can help mitigate the chronic inflammation often associated with obesity and its metabolic disorders (65, 75). Numerous investigations have clearly illustrated a connection between millet usage and lower levels of inflammatory biomarkers, such as interleukins and C-reactive protein, commonly found to be elevated in individuals experiencing obesity (80) Yet, while these biochemical changes are encouraging, it’s important to recognize that inflammatory markers can fluctuate for many reasons, and there are relatively few long-term trials confirming sustained anti-inflammatory benefits of millets alone.

As for thermogenic effects, direct evidence of increased energy expenditure from millet consumption is sparse. In theory, improved lipid and glucose metabolism could boost dietary-induced thermogenesis, which is often impaired in obese individuals (81, 82). Still, without targeted studies measuring thermogenesis before and after adding millets to the diet, any conclusions remain speculative. In general, millets present various pathways for enhancing metabolic health—ameliorating lipid profiles, modulating glycemic levels, fostering satiety, and mitigating inflammation, yet they ought to be regarded as a singular element of a well-rounded dietary regimen Individual responses may vary, and long-term, large-scale research is needed to fully understand how different millet varieties affect obesity-related outcomes. The incorporation of millets into routine dietary regimens is most effective when synergistically combined with consistent physical exercise and additional nutritious eating practices, rather than being perceived as an isolated remedy.

### 4.4 Cancer

Researchers find millet polyphenols exciting because they can reverse the cancer-linked epigenetic tweaks, such as DNA methylation and histone changes, that tend to flip on oncogenes or shut down tumour-suppressor genes (83, 84). By adjusting gene activity at the epigenetic stage, these agents might affect how cancer cells behave and could revert malignant characteristics (83). However, most of the evidence comes from *in vitro* or animal studies, and it remains unclear how effectively millet polyphenols reach target tissues in humans or whether the doses used experimentally can be achieved through a typical diet.

Without a deeper exploration into bioavailability and pharmacokinetic features, the implementation of these discoveries in clinical practice is untimely. Beyond their epigenetic effects, millet polyphenols also slow cancer growth by throwing off the cell cycle and encouraging apoptosis, the body’s own way of clearing out malignant cells (85, 86). There are certain substances that may start apoptosis in malignant cells and hinder the cancer-causing pathway, reducing the size and spread of tumors (86). Although these anti-proliferative properties exhibit considerable potential, numerous investigations are predicated on elevated concentrations of isolated substances that may lack physiological relevance. In addition, neoplastic cell lines do not invariably replicate the intricate tumour microenvironment, suggesting that these findings may overstate the therapeutic efficacy in comparison to what would be observed *in vivo*.

Millet-derived polyphenols show real promise in slowing metastasis by blocking the enzymes MMP-2 and MMP-9, which normally break down the surrounding tissue and let cancer cells spread (87). These agents can instigate the demise of tumor cells and obstruct carcinogenic processes, which results in lessening tumor expansion and metastasis (86). While the anti-proliferative effects appear to be hopeful, many research efforts focus on substantial concentrations of isolated substances that could lack physiological significance. Their anti-inflammatory properties also mitigate chronic inflammation, a condition frequently associated with the advancement of cancer (65). Yet, antioxidant and anti-inflammatory benefits observed in cell culture or animal models may not directly translate into significant clinical outcomes. The concentration and form of polyphenols reaching human tissues can differ markedly from those used in laboratory assays. Although millet-derived polyphenols exhibit encouraging anti-carcinogenic characteristics, it is essential to acknowledge the intricate nature of cancer as a pathological condition and the heterogeneity in individual responses to therapeutic interventions. The efficacy of these bioactive compounds may be affected by variables including bioavailability, metabolic processes, and the particular type of malignancy. Clinical trials in humans are scarce, and without robust, long-term studies, claims about cancer prevention or therapy remain speculative. Additionally, potential synergistic effects when combined with other therapeutic agents or dietary components should be explored further to determine whether millet polyphenols can genuinely enhance efficacy or reduce side effects in combination treatments. Subsequently, millet polyphenols serve as a compelling starting point for research, but translating laboratory findings into practical dietary recommendations or pharmaceutical therapies will require much more rigorous investigation.

Lunasin, a peptide found in millet, has shown promise in cancer prevention by blocking histone acetyltransferases (HATs), the enzymes that add acetyl groups to histones and help control gene activity. *In vitro* studies suggest that lunasin survives digestion and is capable of entering cell nuclei to inhibit HAT activity, hinting at its role in cancer prevention through epigenetic modulation (88). However, these promising results are largely based on cell-culture experiments. It remains unclear whether lunasin reaches sufficient concentrations in human tissues when consumed as part of a normal diet, and whether its activity is maintained in complex physiological environments.

Polyphenol-abundant extracts derived from pearl millet have been documented to facilitate apoptosis in breast carcinoma cells, encompassing those that exhibit resistance to conventional pharmacological treatments, by enhancing cytotoxic autophagy and augmenting markers indicative of apoptosis (89). Encapsulation techniques appear to improve their bioavailability and enhance anticancer effects, suggesting that millet-derived polyphenols could augment existing therapies. While these findings are encouraging, most studies use isolated compounds or specialized delivery systems under controlled conditions. It is still uncertain whether similar benefits would be seen in humans consuming whole millet grains or conventional millet-based foods, where polyphenol concentrations and absorption may vary widely.

The soluble fiber obtained from foxtail millet has demonstrated noteworthy antitumor characteristics in colorectal cancer experiments via the promotion of gut microbiota diversity and changes in cancer-linked signaling pathways (90). This suggests that millet fiber might support colorectal cancer prevention and biotherapy by promoting a healthier gut environment. On the other hand, translating results from animal or ex vivo studies into human recommendations is challenging. Differences in human microbiota composition, dietary patterns, and fiber intake levels mean that the protective effects seen in models may not directly reflect real-world outcomes. Studies indicate that components in millet bran, particularly ferulic acid and p-coumaric acid, can boost the susceptibility of colorectal carcinoma cells to chemotherapy medications like oxaliplatin by affecting multidrug resistance proteins through changes in ganglioside GM3 degradation (91).

Overall, research on millet’s epigenetic and anticancer properties is encouraging but preliminary. Epigenetic modifications such as the methylation of DNA and the modification of histones are indubitably significant in the oncogenic processes of initiation and advancement, and the strategic targeting of these modifications has demonstrated potential efficacy with pharmacological agents, such as ENL inhibitors, that modulate oncogenic transcription (92). These wider insights remind us that cancer is complex and a single dietary component likely cannot replace multifaceted treatment strategies. While millet compounds could become part of a holistic approach to cancer prevention or adjunct therapy, large-scale human trials and a deeper understanding of bioavailability, metabolism, and potential synergies or interactions are essential before making definitive dietary recommendations.

### 4.5 Neurodegenerative diseases

Studies suggest that millet polyphenols can lower oxidative stress in brain cells by boosting antioxidant enzymes, which neutralize reactive oxygen species and protect neurons (93, 94). At the same time, eating millet appears to reduce the activity of Alzheimer’s-related genes like APP, tau, and *γ*-secretase, potentially helping to prevent the buildup of amyloid plaques and tangles that characterize AD (93). While these findings are encouraging, they come primarily from cell-based studies, where concentrations and exposure conditions can be tightly controlled. It remains unclear whether similar effects occur in the human brain, where absorption, metabolism, and complex cell–cell interactions may alter the compounds’ activity.

Compared to various natural antioxidants—such as curcuminoids, ascorbic acid, and tocopherols, millet polyphenols bring specific benefits to the table regarding gene expression modulation and oxidative stress reduction; however, they do not solely account for neuroprotection (95, 96). Many antioxidants operate through similar mechanisms, such as direct scavenging of free radicals or dampening inflammatory pathways. In fact, clinical trials involving curcumin or vitamin E have shown mixed results: some studies suggest modest benefits, while others find no significant effect on disease progression (97, 98). The critical point is that head-to-head comparisons in humans are limited, and single-compound interventions often fail to capture the complexity of neurodegenerative diseases. Therefore, although millet polyphenols look promising in preclinical models, it’s premature to claim that they outperform other antioxidants without rigorous human trials.

A significant challenge faced by any dietary antioxidant, inclusive of those sourced from millet, entails the traversal of the blood–brain barrier (BBB) *In vitro* and rodent models may show neuroprotective effects, but translating those results to humans is challenging because not all polyphenols penetrate the BBB effectively (99). Furthermore, clinical studies on antioxidants in neurodegenerative diseases often yield inconsistent outcomes, sometimes due to suboptimal study design or starting treatment too late in the disease process (97, 99). In light of these limitations, millet-derived antioxidants should be viewed as one piece of a multifaceted strategy—potentially supporting direct neuroprotection and indirect modulation of inflammatory pathways, but not as standalone cures. Ultimately, more comprehensive, well-controlled human research is necessary to clarify their role and optimal use in tackling neurodegenerative disorders. Uric acid (UA) is essential as a primary antioxidant in our bodies, representing a significant percentage of the extracellular antioxidant defense, and it has shown neuroprotective characteristics in the context of Parkinson’s and Alzheimer’s by nullifying free radicals (93). Despite these promising findings, UA therapy comes with significant caveats which is the individual variability in UA metabolism can lead to unpredictable outcomes, and elevated UA levels are associated with gout and cardiovascular risks. These adverse effects limit UA’s clinical application, suggesting that a deeper understanding of its mechanisms and careful dose optimization are essential before it can be reliably used in neurodegenerative settings (100).

Beyond UA, a range of antioxidant therapies is under investigation to reduce oxidative damage in neurodegenerative disorders. Catechins and epigallocatechin-3-gallate (EGCG) originating from green tea—have exhibited substantial antioxidant and anti-inflammatory characteristics, proficiently quelling free radicals and diminishing neuroinflammation (94). The stimulation of the Nrf2–Keap1 signaling mechanism aids in the expression of cytoprotective genes that improve mitochondrial function and reduce inflammation in animal experiments showcasing neurodegenerative (101).

Formulations from Traditional Chinese Medicine (TCM) have also been suggested for their potential to alleviate oxidative stress and neuroinflammation; however, the majority of the corroborative evidence is derived from cellular or clinical animal studies, with high-caliber clinical trials being notably limited (102). Furthermore, oxidative stress can significantly compromise the stability of the blood–brain barrier (BBB), enabling the entry of neurotoxic elements into the central nervous system; methods designed to support BBB integrity are currently in preliminary development and demand stronger validation (103). Moreover, many antioxidant trials have produced inconsistent results—possibly due to late-stage intervention, inadequate bioavailability, or suboptimal dosing (97, 99).

## 5 Development of millet-based functional foods

The production of millet functional food products is increasing because of the nutritional and health benefits linked with millets. The functional versatility of millets makes it possible to integrate them into many food products, from conventional foods to convenience foods. Such versatility, in addition to their health values, makes millets a beneficial ingredient in the production of functional foods. Millets are used in various food products, including baked goods, beverages, and traditional foods like roti and dosa. Modern processing techniques like extrusion and baking have expanded their use in ready-to-eat and ready-to-cook products. Innovative products such as millet-based cakes have been developed.

### 5.1 Flatbreads

Chapati, roti, and rotla are traditional gluten-free breads made from pearl millet flour or fermented pearl millet flour (104). The use of finger millet flour in wheat-based flatbread formulations has been shown to improve nutritional quality and offer functional health benefits. Experimental trials where wheat flour was replaced with 20% extruded finger millet flour have shown that puffing behavior is greatly improved, dough resistance reduced, shrinkage minimized, and baking time decreased compared to its unextruded counterpart. Sensory acceptability tests showed that these products were as acceptable as normal whole wheat flatbread (105). Maize-gluten-free flatbreads containing 10%–30% partially substituted extruded finger millet flour have improved rheological properties, functional attributes, and nutritional patterns. The blend of composite compositions has structural strength while at the same time also improving the final product’s functionality (106).

Composite flatbreads prepared with 3:1 proportion of refined wheat to millet flour show greater baking loss and shrinkage, less puffing, and lower acceptability as judged by senses in comparison to their single pure wheat-based counterparts (107). Doughs with 20% finger millet flour inclusion exhibit good handling and sheeting characteristics, yielding chapatis of acceptable quality in texture and nutritional value. However, sensory acceptability decreases when the level of inclusion exceeds this value. Despite the reduced consumer acceptability of millet-based flatbreads—mainly due to their distinctive sensory and textural properties, the use of millet is still a viable strategy for improving the techno-functional quality and dietary incorporation of underutilized cereal grains. Finally, thermal treatment of chapatis seems to lead to a decrease in their antioxidant activity compared to the raw flour, indicating some degradation of the bioactive compounds during cooking (108).

### 5.2 Cookies

Cookies are conventionally prepared using a mixture of flour, fat, and sugar, and new formulations have also investigated the use of millets as functional, gluten-free ingredients. In a study Sharma et al. (109), germinated foxtail, barnyard, and kodo millet flours were combined in a 70:20:10 ratio to develop composite flour cookies. This specific combination showed desirable sensory properties and enhanced nutritive value, particularly with regard to fiber and micronutrient availability. Awolu et al. (110) investigated cookies prepared from diverse flour sources, including soybean, rice, millet, and tiger nut, which underwent treatments such as fermentation, malting, and debranning. Among these, blends containing fermented millet yielded the highest sensory scores, highlighting the value of pre-processing in enhancing palatability and functionality.

Another study by Hussain et al. (111) evaluated the progressive substitution of wheat flour with millet flour in cookie formulations. While antioxidant activity increased with greater millet content, samples containing more than 50% millet were less favoured in terms of taste and texture. These findings indicate that blending millet flour with wheat in appropriate proportions can produce cookies with improved functional properties while maintaining consumer acceptability.

### 5.3 Alcohol and non-alcohol beverages

Millets serve as valuable raw materials in the development of plant-based beverages due to their inherent health benefits and gluten-free nature. It is a rich source of dietary fiber and polyphenols, unprocessed millet contributes to the nutritional profile of both alcoholic and non-alcoholic drinks.

Millet based beverages such as Malwa, Bantu, Pombe, and Opaque(kafir) beer are widely produced across various regions. Jandh, a traditional alcoholic drink from Nepal, is prepared using finger millet (112) Similarly, Kunun-zaki is a popular non-alcoholic fermented beverage made from a mixture of millet, maize, sorghum, spice and sugar. Another notable example is Mbege ale, a traditional beer derived from millet, sorghum and banana, which has been industrially processed and commercialized under the name Chibuku Shake. This beverage retains significant popularity, especially in countries such as Botswana, Zambia, and Zimbabwe (113).

### 5.4 Millet based probiotic and prebiotic products

Prebiotics are characterized as non-digestible dietary components that selectively enhance the proliferation and functionality of beneficial colonic microbiota, thus fostering host health (114) documented the extraction of a distinctive polysaccharide from millet that exhibited resistance to enzymatic hydrolysis by salivary and pancreatic *α*-amylases. This polysaccharide further demonstrated significant prebiotic effectiveness, as evidenced by its activity score with *Lactobacillus acidophilus* and *L. brevis.*

The formulation of functional beverages that integrate both prebiotic and probiotic components has attracted scholarly interest. An optimized white finger millet probiotic beverage (OWMPB) was developed utilizing 2% pineapple crown powder (PCP) and 1% (v/v) *Lactobacillus rhamnosus* GG (LGG, NCDC 347) (115). Probiotics are defined as live microorganisms that, when ingested in sufficient quantities, confer health benefits to the host, predominantly through the modulation of the gut microbiota (39). Malini et al. (116) successfully engineered a plant-based probiotic beverage that incorporated pineapple core powder and *Lacticaseibacillus rhamnosus* (LGG). The optimized formulation—designated as PAMPB, comprised white finger millet, sugar, PCP, and LGG in a proportional ratio of 14:5:2:2, respectively (117).

These findings collectively underscore the potential of millets as a viable substrate for the innovation of functional beverages endowed with both prebiotic and probiotic properties.

## 6 Millet and sustainable food systems

Millets have been found to be climate-resilient crops with very good chances of relieving challenges arising due to global climate change. Agroclimatic projections in the future—involving increased temperatures of 2–5 °C, decreased rainfall, increased water scarcity, and heightened malnutrition threats are likely to adversely affect traditional cropping systems. In contrast to rice and wheat, millets possess better fitness to these unfavourable conditions. Flooded rice cultivation is a principal source of methane emissions and, therefore, climate-stress unsustainability. Wheat, being thermosensitive, is susceptible to yield loss with rising temperatures and may become less suitable on arable land. Compared to this, millets are drought-resistant, low-input crops that sequester carbon and help in climate change mitigation. They provide diverse co-benefits in addition to food security in terms of increased nutritional content, as animal fodder, better public health, rural livelihood support, and environmental sustainability. Due to their multifunctionality, they are strategic crops to use in enhancing agricultural resilience and ensuring long-term sustainable areas (118).

Being traditional grains in India, millets make a vital contribution to national food security, especially among rural and economically poor people. Their potential to grow in harsh agroclimatic conditions, with low input use and suitability to marginal lands, makes them best suited for smallholder and resource-poor farmers. Their longer shelf life also contributes to the increased availability of food during lean times, thereby increasing the resilience of food systems. With their drought resistance, low cost, and low agronomic requirements, millets are a sustainable livelihood choice under economic and environmental stress. Their local availability and low price make them key assets for sustaining food and nutrition security during socio-economic shocks and climate extremes. Encouraging millet cultivation is aligned with strategic goals for sustainable agriculture, climate adaptation, and inclusive food system resilience (119).

## 7 Challenges and future directions

Millets are often perceived as less used compared to more commonly consumed grains like rice and wheat a stigma rooted in their traditional image as “poor people’s food,” which unfairly deters middle and upper-class consumers. Market shelves scarcely feature high-quality millet grains or processed items, a direct consequence of underdeveloped supply chains and inadequate infrastructure that leaves potential buyers with less choice (120–122). Despite mounting evidence of their rich vitamin, mineral, and fiber content, consumers remain largely oblivious to these benefits—a failure compounded by feeble educational campaigns (123–125). Conventional extraction methods for bioactive compounds in millets remain frustratingly energy-intensive and prone to thermal degradation, highlighting an urgent need to move beyond outdated practices (126); while green-assisted techniques like ultrasound and microwave extraction appear promising for faster, more efficient yields, it is puzzling that their application to minor millets is still not optimized despite widespread acclaim (126). Enzyme-assisted extraction, which could circumvent high energy inputs and better handle complex matrices, has nonetheless been largely overlooked in millet-specific contexts and deserves far deeper investigation (126). While we know that finger millet has phytochemicals like ferulic acid and quercetin that are bioaccessible, the move from bioaccessibility to real bioactivity and health gains remains hypothetical until more extensive research is pursued (127).

Molecular docking studies have hinted that flavan-4-ol and other millet-derived compounds could inhibit lifestyle diseases, but instead of resting on these in silico findings, the field must urgently prioritize clinical validation (128). Similarly, although simulation studies suggest millets might aid in obesity management, declaring therapeutic potential without extensive *in vitro*, *in vivo*, and human trials borders on wishful thinking (78). Claims regarding millets in COVID-19 prevention also remain inconclusive, underscoring the necessity of well-designed randomized controlled trials before making any public health recommendations (129). On the breeding front, the call to leverage genetic resources to develop millet varieties with superior nutrient profiles and climate resilience is sensible, but the pace of progress is disappointingly slow given the escalating need for sustainable crops (130). There are many important gaps despite millets’ potential health benefits. Standardised and improved extraction protocols are essential for the reliable isolation and characterisation of bioactive compounds across studies. The bioavailability, metabolism, and tissue distribution of these compounds in humans are inadequately understood, preventing the development of effective dietary treatments. The majority of existing data derives from *in vitro* and animal studies, while comprehensive, large-scale randomised human trials evaluating efficacy, optimal dosage, long-term safety, and disease-specific results are absent. The comparability of research findings is further affected by the variety of millet species, processing methods, and dietary formulations. Identifying these gaps through interdisciplinary research will be crucial in delivering evidence-based recommendations and effectively incorporating millets into present dietary and medical procedures.

Governments have implemented policies such as financial assistance for millet cultivators, incentives for products derived from millet, and the incorporation of millet into public distribution frameworks (131, 132); however, in practice, these initiatives frequently fail to adequately benefit smallholder farmers and tend to favor those producers with better networking capabilities. The International Year of Millets 2023 has catalyzed notable international cooperation and the sharing of best practices (133), yet it remains uncertain whether this momentum will yield sustainable long-term support. Institutional initiatives that promise advancements in seed varieties and agricultural technologies (134) appear encouraging in theory, although numerous farmers continue to encounter difficulties in accessing high-quality inputs. Millets are unequivocally abundant in proteins, carbohydrates, dietary fibers, essential fatty acids, vitamins (notably B vitamins), and minerals (including iron, zinc, magnesium, and calcium) (134–136); however, it is indeed perplexing that their esteemed status as a nutritional powerhouse has not manifested in broader societal acceptance Despite their benefits, millets remain underutilized due to limited awareness, lack of infrastructure, and policy neglect (65, 136). Technological advancements, such as multi-OMICS strategies, can enhance the nutritional traits of millets, making them more appealing to consumers (137), but without sustained investment and public engagement, such innovations risk gathering dust.

## 8 Conclusion

In conclusion, this review highlights the evidence that millets play an important role as functional foods in preventing and managing chronic lifestyle diseases. Millets, characterized by their varied nutrient composition, plentiful bioactive constituents, and glycemic characteristics, make them a dietary option for mitigating the escalating prevalence of CLDs on a global scale. The review investigates underexplored yet increasingly pertinent areas, such as the interaction of millet-derived fibers and polyphenols with the gut microbiota, their neuroprotective and epigenetic influences, and the significance of millets within personalized nutrition paradigms. Moreover, the incorporation of millets into sustainable food systems yields dual advantages, nutritional security and environmental resilience, especially in light of climate change and resource limitations. Notwithstanding these encouraging attributes, considerable challenges remain, including insufficient consumer awareness, taste preferences, and the necessity for rigorous human clinical trials to validate mechanistic understandings. Consequently, future research should embrace a multidisciplinary and translational framework, integrating advancements in food science, genomics, microbiology, agronomy, and public health. Improving the implementation of new policies, innovation in millet-based food products, and increasing public awareness through nutrition education are essential to move millets from traditional staples to health-promoting diets. Ultimately, millets have great potential to support sustainable and effective dietary approaches for preventing and managing chronic lifestyle diseases.
